# Concurrent expression of *HP-NAP* enhances antitumor efficacy of oncolytic vaccinia virus but not for Semliki Forest virus

**DOI:** 10.1016/j.omto.2021.04.016

**Published:** 2021-05-05

**Authors:** Jing Ma, Chuan Jin, Matko Čančer, Hai Wang, Mohanraj Ramachandran, Di Yu

**Affiliations:** 1 Department of Immunology, Genetics and Pathology, Science for Life Laboratory, Uppsala University, 75185, Uppsala, Sweden; 2 CAS Key Laboratory for Biomedical Effects of Nanomaterials and Nanosafety, CAS Center for Excellence in Nanoscience, National Center for Nanoscience and Technology, Beijing 100190, China; 3 University of Chinese Academy of Sciences, Beijing 100049, China

**Keywords:** oncolytic virus, Semliki Forest virus, vaccinia virus, NAP, neutrophil-activating protein, immunotherapy, GD2, neuroblastoma

## Abstract

Oncolytic viruses (OVs) represent promising therapeutic agents for cancer therapy by selective oncolysis and induction of anti-tumor immunity. OVs can be engineered to express tumor-associated antigens and immune-modulating agents to provoke stronger antitumor immunity. Here, we engineered vaccinia virus (VV) and Semliki Forest virus (SFV) to express neuroblastoma-associated antigen disialoganglioside (GD2) and the immune modulator *Helicobacter pylori* neutrophil-activating protein (NAP) and compared their therapeutic potency. Oncolytic VV did not exhibit any antitumor benefits, whereas SFV was able to delay subcutaneous neuroblastoma (NXS2) tumor growth. Additional expression of the GD2 mimotope (GD2m) by VV-GD2m or SFV-GD2m did not improve their anti-tumor capacity compared to the parent viruses. Further arming these OVs with NAP resulted in contrasting anti-tumor efficacy. VV (VV-GD2m-NAP) significantly improved therapeutic efficacy compared to VV-GD2m, which was also associated with a significantly elevated anti-GD2 antibody, whereas there was no additive antitumor efficacy for SFV-GD2m-NAP compared to SFV-GD2m, nor was the anti-GD2 antibody response improved. Instead, NAP induced higher neutralizing antibodies against SFV. These observations suggest that distinct immune stimulation profiles are elicited when the same immunostimulatory factor is expressed by different OVs. Therefore, careful consideration and detailed characterization are needed when engineering OVs with immune-modulators.

## Graphical Abstract

**Figure:**
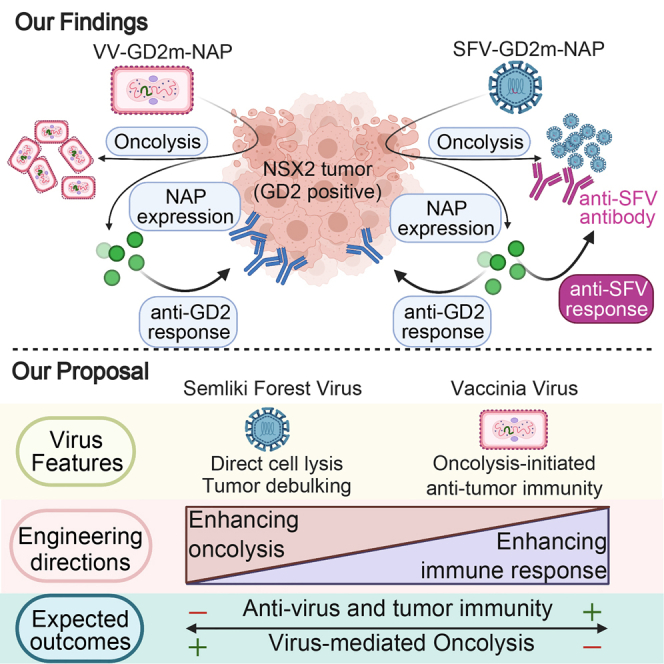

## Teaser

*Helicobacter pylori* neutrophil-activating protein (NAP) enhanced the therapeutic effect of oncolytic vaccinia virus expressing tumor-associated antigen (TAA) via inducing humoral response against the TAA. In contrast, NAP did not improve oncolytic Semliki Forest virus efficacy in a similar setting due to polarization toward the anti-virus immune response instead.

## Introduction

Oncolytic viruses (OVs) are promising agents for cancer therapy due to their ability to selectively replicate in and destroy tumor cells while leaving the healthy cells unharmed. To date, a variety of viruses have been investigated as prospective cancer therapeutic agents, ranging from small RNA viruses to large DNA viruses.^1^, ^2^, ^3^, ^4^ The oncolysis of tumor cells can be immunogenic and can elicit an anti-tumor immune response, contributing to the OV therapeutic effects to a large extent.^2^^,^^5^ Further enhancing the anti-tumor immune response is one way to improve its efficacy. We constructed oncolytic Vaccinia virus (VV) and Semliki Forest virus (SFV) to express tumor-associated antigen (TAA) and immune modulator and investigated their ability to induce an anti-tumor immune response in this study.

VV was used as a vaccine against *Variola*, the causing virus of smallpox, and then developed also as a cancer vaccine or OV.^6^^,^^7^ It is an enveloped DNA virus, replicates in the cytoplasm. The virus has broad applicability due to its broad tropism^8^^,^^9^ and can spread efficiently between tumor cells via cell-cell junctions and metastatic lesions through the bloodstream.^10^^,^^11^ VV can also accommodate various transgenes due to its large coding capacity.^12^ Recent clinical studies also approve its safety and tumor-selected replication.^6^^,^^13^ We used strain Western Reserve (WR) with thymidine kinase (TK) deletion (VV-dTK) as an oncolytic VV in this study,^14^ as the virus is more virulent in animal models. Deletion of TK (dTK) renders the virus replication preferentially in dividing cells, which favors tumor targeting and selection.^15^

SFV is an enveloped, positive-strand RNA virus belonging to the *Togaviridae* family, and it has a broad host range that could infect and kill a variety of tumor cells.^16^ The virus has a natural capability to penetrate the blood-brain barrier in mice and made it a perfect candidate for targeting brain and other neurological tumors. Strain L10 and its lab derivative SFV4 are virulent in mice by causing brain encephalitis, whereas the SFVA7(74) strain used in this study is an avirulent strain that carries several attenuating mutations within the nonstructural open reading frame.^17^ Researchers, including us,^3^ have developed this strain as an oncolytic agent and evaluated it in various tumor models.^18^^,^^19^

Disialoganglioside (GD2) is a well-characterized, neuroblastoma-associated glycolipid antigen, and Dinutuximab, a GD2-binding antibody, is approved for treatment of neuroblastoma. Here, we engineered VV and SFV with TAA by expressing a reported GD2 mimotope (GD2m), which can structurally mimic GD2 and induce an anti-GD2 antibody response when delivered as a plasmid expression vector.^20^

Neutrophil-activating protein (NAP) of *Helicobacter pylori* bacteria is a small dodecameric protein and acts as a major virulence factor.^21^ NAP is a chemoattractant and activator of neutrophils, monocytes, and dendritic cells (DCs) mainly by Toll-like receptor (TLR)-2 stimulation. Our previous studies also showed that NAP has the potential to drive T helper cell type 1 (Th1) polarization by creating an interleukin (IL)-12- and IL-23-enriched milieu.^22^ Arming an oncolytic adenovirus with NAP improved efficacy in an immune-deficient animal model.^23^ Further, NAP also boosts the antigenicity of weak immunogens when co-expressed together.^24^ These findings encouraged us to further study whether OV-expressed TAAs can be tailored with NAP to boost its immunogenicity.

In this study, we hypothesize that the therapeutic efficacy of OVs can be further improved by co-expression of TAA and NAP. With the use of an NXS2 neuroblastoma tumor model, we demonstrate that arming NAP (SFV-GD2m-NAP) adds no improvement to oncolytic SFV when compared with the non-modified SFV (SFV-GD2m), instead the anti-SFV antibody response was boosted by NAP. On the other hand, VV-GD2m-NAP significantly reduced tumor growth and prolonged mice survival in comparison to its parental virus (VV-GD2m). This was associated with an enhanced anti-GD2 antibody response rather than the anti-VV antibody response.

## Results

### The oncolytic VV-GD2m and SFV-GD2m express GD2m

**Figure 1:**
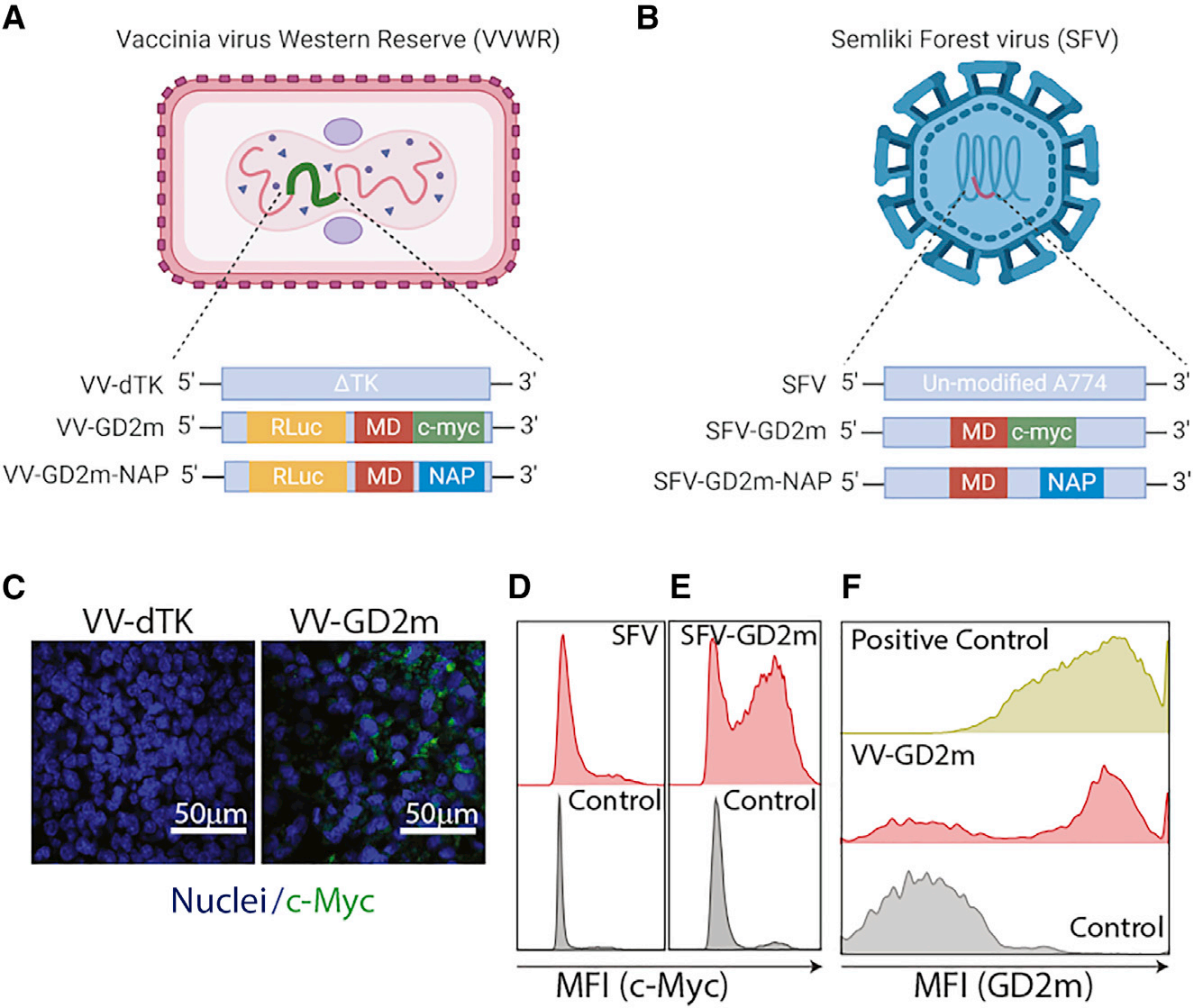
Oncolytic Vaccinia virus (VV) and Semliki Forest virus (SFV) encoding the GD2 mimotope (GD2m) (A and B) Schematic illustration of VV Western Reserve (VVWR) and SFV constructs used in the study. (C) GD2m expression in NXS2 tumors injected with VV-dTK or VV-GD2m (i.t.), visualized by immunohistochemical staining. Tumors were collected 2 days after virus injection, and the tumor tissue section was stained with anti-c-Myc antibody and DAPI. Scale bars, 50 μm. (D and E) GD2m expression in NXS2 tumors injected with SFV or SFV-GD2m (i.t.). Tumors were harvested 2 days after virus injection and single-cell suspension was prepared and stained with anti-c-Myc antibody. Representative histogram figures were shown. (F) Representative histogram showing binding of the anti-GD2 antibody to cells infected with VV-GD2m. B16-F10 cells were infected with VV-GD2m (MOI 0.1) and were collected after 48 h. Cells were stained with anti-GD2 antibody (clone 14G2a) and secondary goat anti-rabbit-AF647 antibody and analyzed by flow cytometry. NXS2 cells (naturally expressing GD2) were used as a positive control. VV-GD2m-infected B16-F10 cells stained with only secondary goat anti-rabbit-AF647 antibody were used as a negative control.

To target neuroblastoma-associated antigen GD2, we engineered oncolytic VV and SFV to express a peptide GD2m. In VV constructs, the mimotope was fused with a *Renilla* luciferase (Rluc) and c-Myc tag, in the backbone of a tumor-selective VV-dTK, in which TK has been deleted (Figure 1A). In SFV constructs, the mimotope was fused with a c-Myc tag alone (Figure 1B). We first confirmed the expression of mimotope by VV-GD2m or SFV-GD2m upon infection of established tumors *in vivo* (Figures 1C−1E). Furthermore, we observed that viral-expressed GD2m can be recognized by the anti-GD2 antibody 14G2a (Figure 1F), indicating the design is feasible. To demonstrate that GD2 is a suitable target antigen and can be vaccinated against by using GD2m, we first immunized mice using an adenoviral vector expressing GD2m and challenged these mice with GD2-positive NXS2 cells. The tumor growth was significantly delayed in immunized mice compared to unimmunized mice (Figure S1), indicating the feasibility of using GD2 as a model antigen for the current study.

### Arming OVs with mimotope does not affect the virus infection and replication *in vitro* and *in vivo*

**Figure 2:**
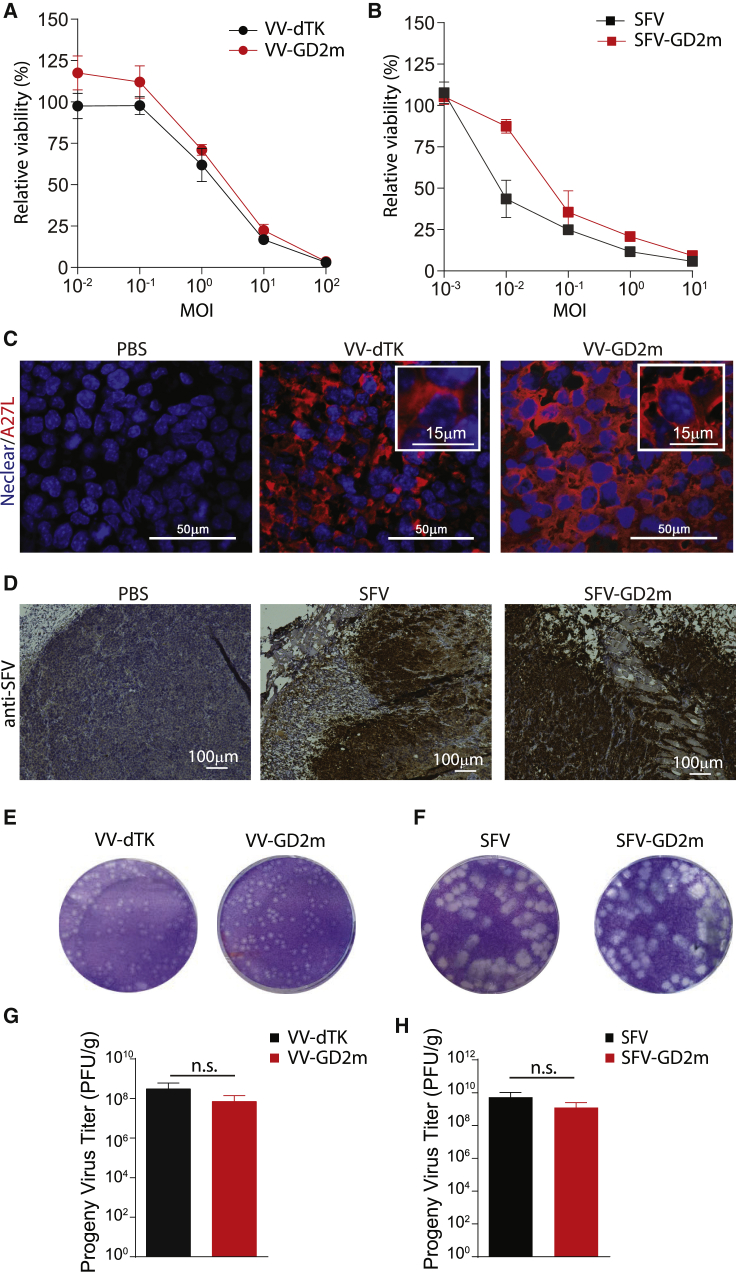
Replication and oncolysis capacity of GD2m-expressing OVs both *in vitro* and *in vivo* (A and B) Relative cell viability of NXS2 murine neuroblastoma cells after infection with either VV (VV-dTK or VV-GD2m, MOI 0.01 to 100) or SFV (SFV or SFV-GD2m, MOI 0.001 to 10). Value was measured 4 days post-infection (d.p.i.) and presented (mean ± SEM) as percentage relative to non-infected control cells. (C) The spread of VV-dTK and VV-GD2m in the established NXS2 tumor after i.t. injection. VV was visualized by staining vial A27L protein (red), and nuclear was visualized with DAPI staining (blue). Scale bars, 50 μm. (D) The spread of SFV or SFV-GD2m in established NXS2 tumor after i.t. injection. SFV was stained using polyclonal rabbit anti-SFV antibody and HRP-conjugated anti-rabbit antibody and visualized with chromogen DAB. Scale bars, 100 μm. (E and F) Representative plaque morphology of the parental and GD2-armed viruses 72 hours post-infection (h.p.i.) (G and H) Virus titer from tumor lysates 3 d.p.i. quantified using a plaque assay. Values were normalized to tumor weight and compared using parametric t test (n.s., no statistical significance).

To determine if the insertion of transgenes affects virus killing capacity and virus spreading ability in solid tumors, we examined VV-GD2m and SFV-GD2m both on *in vitro*-cultured NXS2 cells and subcutaneous (s.c.)-established *in vivo* tumors. NXS2 cells were infected with viruses at various multiplicities of infection (MOIs) as indicated, and relative cell viability was measured on day 4. Both VV-GD2m and SFV-GD2m showed the killing of NXS2 cells in a dose-dependent manner, and the efficacy was similar to their corresponding parental viruses VV-dTK and SFV (Figures 2A and 2B).

To evaluate viruses’ replication *in vivo*, s.c. NXS2 tumor was established on A/J mice and treated intratumorally (i.t.) with either engineered or parental viruses. Similar staining intensity for viral proteins in the established tumor was observed between engineered and parental viruses (Figures 2C and 2D). We also titrated the progeny viruses recovered from these treated tumors. Similar plaques in size and morphology were formed between engineered and parental viruses (Figures 2E and 2F). Besides, similar viral titers were recovered from the tumors injected with either engineered virus or their parental virus (Figures 2G and 2H). To note, 100 times higher titer was recovered from SFV-treated tumors compared to VV-treated tumors, indicating a faster replication of SFV than VV. This was also reflected by the plaque size in which SFV generated significantly bigger plaques than VV (Figures 2E and 2F).

Taken together, these results indicated that transgene insertion does not affect virus oncolytic activity, replication, and spreading. In addition, SFV showed faster replication and spreading than VV for both parental virus and engineered virus.

### GD2m-armed OVs did not exhibit any therapeutic benefit compared with their parental OVs

**Figure 3:**
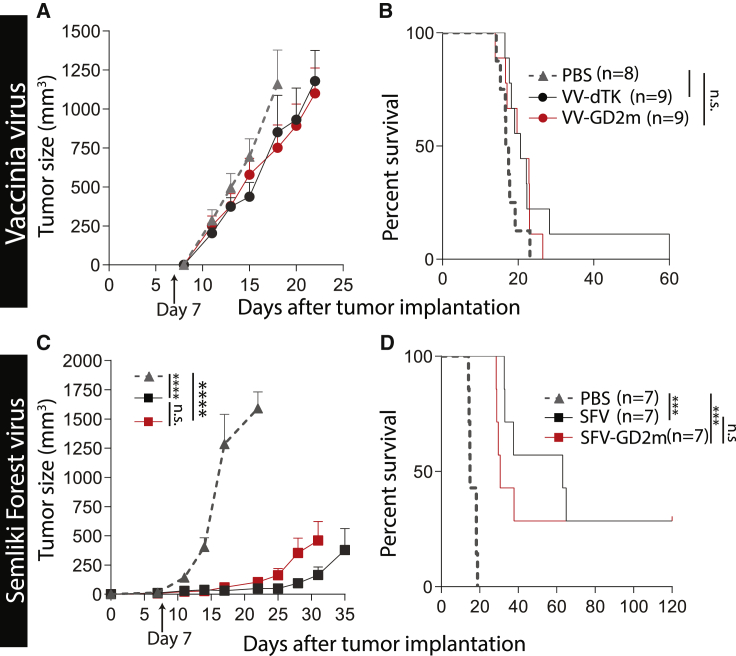
Therapeutic evaluation of GD2m-expressing OVs (VV-GD2m or SFV-GD2m) (A and B) A/J mice bearing a murine neuroblastoma NXS2 tumor were i.t. treated with either PBS or corresponding viruses as indicated on day 7 after tumor implantation. (A–D) The tumor sizes (mean + SEM) (A and C) and Kaplan-Meier survival curves (B and D) were shown. Tumor sizes between each treatment group were compared using two-way ANOVA with Tukey correction, and survival curves were compared using the log-rank test (∗∗∗p < 0.001).

The therapeutic efficacies of mimotope-armed VV and SFV were tested in a syngeneic NXS2 murine neuroblastoma model. A s.c. NXS2 tumor was established in A/J mice and followed by i.t. OV treatment. Tumors grew rapidly in both VV-dTK- and VV-GD2m-treated mice, similar to PBS-treated mice (Figure 3A). This is reflected in the survival, as no statistical survival benefit was observed upon VV treatment compared to PBS treatment (Figure 3B). On the other hand, tumor growth after SFV treatment was significantly delayed (Figure 3C), and a significant survival benefit was observed in both SFV- and SFV-GD2m-treated mice (Figure 3D). All survived mice remained tumor free for 120 days before they were euthanized. However, SFV-GD2m did not show improved therapeutic efficacy over treatment with SFV on NXS2 tumor-bearing mice (Figures 3C and 3D). Therefore, the expression of mimotope by both VV and SFV did not contribute to therapeutic efficacy.

### GD2m-armed OVs did not induce a stronger anti-GD2 humoral immune response

**Figure 4:**
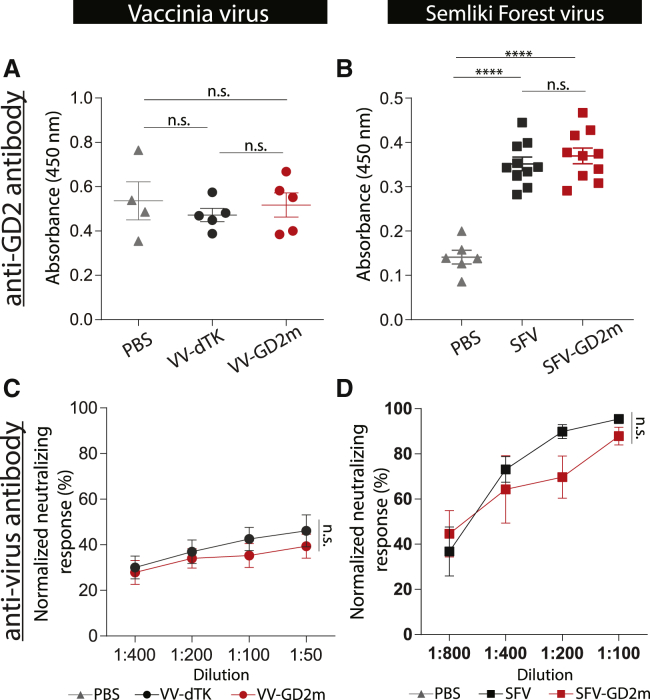
Humoral response against GD2 and viruses induced by GD2m-expressing OVs The serum was collected from NXS2-bearing mice treated with PBS, VVs (VV-dTK or VV-GD2m), or SFVs (SFV or SFV-GD2m) for antibody measurement. (A and B) Anti-GD2 antibody level is shown as absorbance determined by ELISA (mean ± SEM). One-way ANOVA with Bonferroni correction was used to compare the mean values between each group (∗∗∗∗p < 0.0001). (C and D) Virus-specific neutralizing antibody measured in the serum. VV-GD2m or SFV-GFP virus was incubated with serially diluted serum as indicated and applied on 911 cells. Renilla luciferase activity (VV) or percentage of GFP-positive cells (SFV) was quantified. Normalized neutralizing response was presented and shown as mean ± SEM. Groups were compared using one-way ANOVA with Bonferroni correction.

GD2m peptides have been shown to efficiently induce a humoral anti-GD2 response.^20^^,^^25^ We then looked for anti-GD2 antibodies in the serum, harvested 2 weeks after OV treatment. There was no increase of anti-GD2 immunoglobulin G (IgG) level in the serum of VV-dTK-treated mice, in comparison to PBS-treated mice (Figure 4A). On the other hand, the average titer of anti-GD2 IgG in SFV-treated mice was 2.5-fold higher than those of PBS-treated mice (Figure 4B). However, OVs expressing GD2m did not enhance the anti-GD2 antibody response, as a similar antibody titer was observed in mice treated with either the engineered virus or the parental OVs (Figures 4A and 4B). Besides, we observed that the neutralizing anti-viral antibody response was elicited after oncolytic viral treatment, likely also due to the oncolysis events (Figures 4C and 4D).

### Concurrent expression of NAP improved the antitumor efficacy of VV-GD2m but not SFV-GD2m

**Figure 5:**
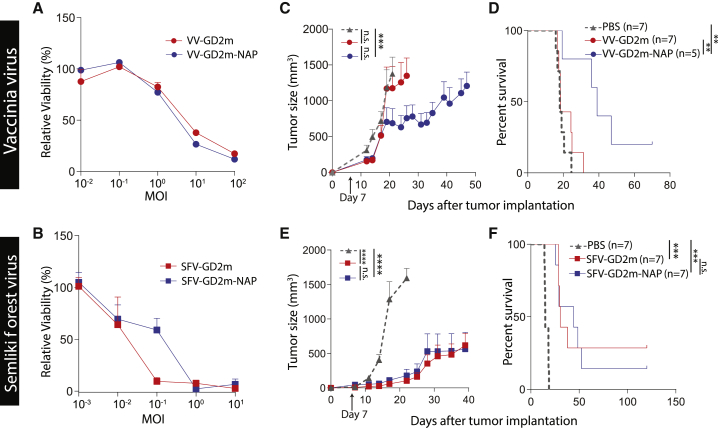
Therapeutic evaluation of NAP-armed OVs (VV-GD2m-NAP or SFV-GD2m-NAP) (A and B) NXS2 cell viability after infection with VVs (VV-GD2m or VV-GD2m-NAP, MOI 0.01 to 100) or SFVs (SFV-GD2m or SFV-GD2m-NAP, MOI 0.001 to 10) on day 4. Cell viability is presented as percentage (mean + SEM) relative to non-infected cells. (C−F) The A/J mice-bearing murine neuroblastoma NXS2 tumor was i.t. treated with either PBS or corresponding OVs as indicated on day 7 after tumor implantation. Tumor sizes (mean ± SEM) (C and E) and Kaplan-Meier survival curves (D and F) are shown. Tumor sizes between each treatment group were compared using two-way ANOVA with Tukey correction, and survival curves were compared using the log-rank test (∗p < 0.05, ∗∗p < 0.01, ∗∗∗p < 0.001, ∗∗∗∗p < 0.0001).

We then hypothesize that further arming the immunogen with NAP might augment the response, since NAP is known to enhance immunogenicity of weak antigens.^24^ We engineered VV-GD2m-NAP and SFV-GD2m-NAP and confirmed that the NAP-armed viruses can kill *in vitro*-cultured cells at similar efficiency in comparison to their parental viruses (Figures 5A and 5B). NXS2-tumor-bearing mice were i.t. treated with different viruses 7 days after tumor implantation. Encouragingly, VV-GD2m-NAP controlled tumor growth (Figure 5C) and prolonged survival (Figure 5D) compared to VV-GD2m treatment. Following the above results, VV-GD2m did not show any therapeutic benefit as compared to PBS control. In addition, arming VV with NAP alone (VV-NAP) did not have any therapeutic benefit compared to PBS control (Figure S2). On the other hand, arming SFV with NAP did not have any benefit in terms of tumor growth control or survival in comparison to its parental virus (Figures 5E and 5F). Taken together, the data suggested in the neuroblastoma tumor model, only oncolytic VV, but not SFV, benefited from NAP arming as an immuno-modulator.

### VV-GD2m-NAP elicited an anti-GD2 antibody response, whereas SFV-GD2m-NAP instead elicited a stronger anti-SFV antibody response

**Figure 6:**
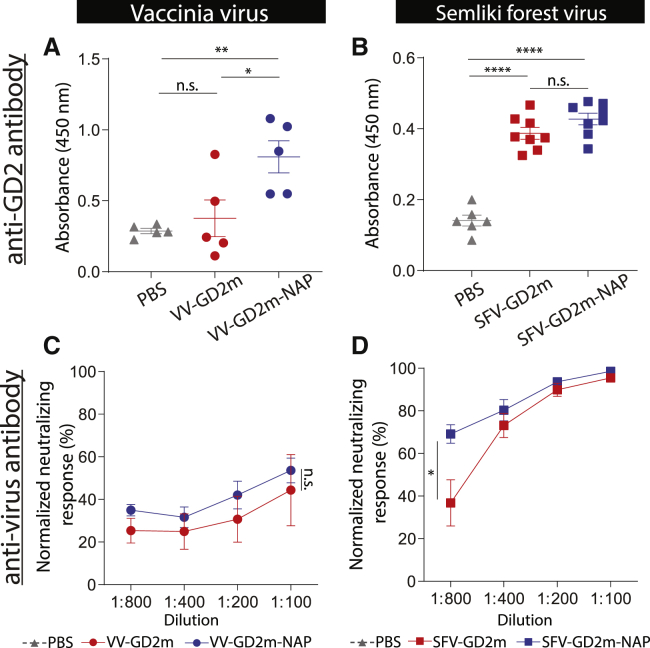
Humoral response against GD2 and viruses induced by NAP-armed OVs The serum was collected from NXS2-bearing mice that received PBS, VV (VV-GD2m or VV-GD2m-NAP), or SFV (SFV-GD2m or SFV-GD2m-NAP) treatment. (A and B) Anti-GD2 antibody level is shown as absorbance determined by ELISA (mean ± SEM). One-way ANOVA with Bonferroni correction was used to compare the mean values between each group (∗p < 0.05, ∗∗p < 0.01, ∗∗∗∗p < 0.0001). (C and D) Virus-specific neutralizing antibody response was measured in the serum. VV-GD2m or SFV-GFP virus was incubated with serially diluted serum as indicated and applied on 911 cells. Renilla luciferase activity (VV) and the percentage of GFP-positive cells (SFV) were quantified. Normalized neutralizing response is presented (mean ± SEM). Groups were compared using one-way ANOVA with Bonferroni correction (∗p < 0.05).

To dissect the differences in therapeutic efficacy between NAP-armed VV and SFV, we investigated the induced anti-GD2 antibody response in serum 2 weeks after OV treatment. VV-GD2m-NAP treatment resulted in a significantly higher anti-GD2 IgG titer, which was 2.5 times higher than VV-GD2m treatment and 4 times higher than PBS control (Figure 6A). In contrast, SFV-GD2m-NAP induced an equal level of anti-GD2 IgG as SFV-GD2m, whereas both were approximately 2-fold higher than the PBS-control group (Figure 6B).

Since OVs are very immunogenic, NAP might skew the antibody response toward anti-virus immunity, which is unfavorable in the cancer therapeutic setting. We next investigated the serum-neutralizing antibody level after virus treatment. Both VV-GD2m and VV-GD2m-NAP induced VV-specific neutralizing antibodies at a similar level (Figure 6C). However, SFV-GD2m-NAP induced a significantly higher titer of SFV-specific neutralizing antibody than SFV-GD2m (Figure 6D). To note, almost 100% of SFV was neutralized by serum at lower dilutions (1:100) when only 40% of VV was neutralized, suggesting that a more potent anti-SFV antibody response was elicited in the mice (Figures 6C and 6D).

**Figure 7:**
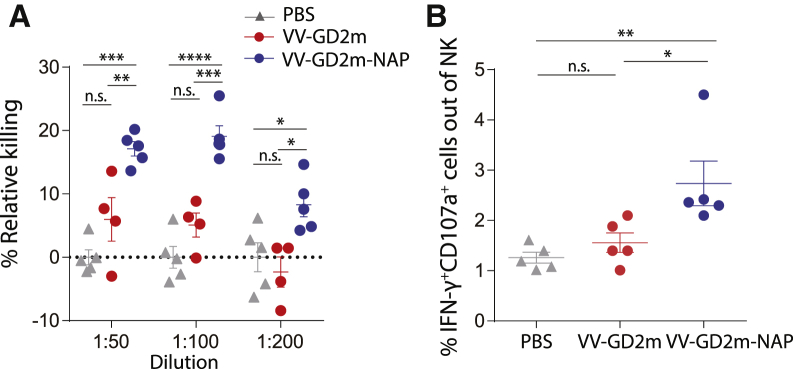
Antibody-dependent cellular toxicity induced by serum from VV-GD2m-NAP-treated mice (A) Relative killing of NXS2 tumor cells in the presence of serum harvested from PBS-, VV-GD2m-, or VV-GD2m-NAP-treated mice (mean ± SEM). One-way ANOVA with Bonferroni correction was used to compare the mean values between groups with each dilution (∗p < 0.05, ∗∗p < 0.01, ∗∗∗p < 0.001, ∗∗∗∗p < 0.0001). (B) The percentage of IFN-γ^+^CD107a^+^ NK cells (gated as CD3^−^NKp46^+^) in the ADCC assay, in the presence of serum isolated from PBS-, VV-GD2m-, or VV-GD2m-NAP-treated mice. (mean ± SEM). One-way ANOVA with Bonferroni correction was used to compare the mean values between groups (∗p < 0.05, ∗∗p < 0.01).

To further investigate the function of the anti-GD2 antibody induced by different VV treatment, we performed an *in vitro* antibody-dependent cell cytotoxicity (ADCC) assay, where naive mice splenocytes were co-cultured with NXS2 tumor cells in the presence of sera harvested from different VV treatment groups. We observed that NXS2 tumor cells were killed more efficiently in the presence of sera harvested from VV-mGD2-NAP compared to other groups (Figure 7A). A significantly higher level of interferon (IFN)-γ^+^CD107a^+^ cytotoxic natural killer (NK) cells was also observed in the co-culture where sera obtained from VV-mGD2-NAP-treated mice (Figure 7B). These results indicated that the anti-GD2 antibody raised in the VV-mGD2-NAP treatment group demonstrated higher ADCC, which further confirmed that an anti-GD2 antibody response contributes to the therapeutic outcome.

In conclusion, concurrent expression of NAP in VV promotes humoral immunity against the co-expressed immunogen, which contributed to the therapeutic outcome, whereas similar construction in SFV-based OV generates a higher anti-viral response, without any additional anti-tumor response.

## Discussion

A variety of OVs are being investigated as prospective cancer therapeutic agents, and recently the focus has shifted to improving the OV-induced anti-tumor immune response. We have previously characterized VV- and SFV-mediated immunogenic cell death of tumors and its ability to induce anti-tumor immune response.^26^ Here, we investigated if the OV-mediated anti-tumor immune response can be further improved by arming it with TAA (GD2) and a pluripotent immune modulator (NAP).

The kinetics of virus replication depends on several factors. Generally, RNA viruses, such as Reovirus, SFV, and Newcastle Disease virus (NDV), kill and replicate in tumor cells faster than DNA viruses (e.g., adenovirus, VV), possibly due to their relatively small genome size and cytoplasmic replication cycle.^27^ At low MOI = 0.1, SFV killed >70% of NXS2 cells, whereas VV barely had any cytotoxicity when assessed at the same time (5 days after infection) (Figures 2A and 2B). This indicated that SFV had faster oncolysis than VV, which is probably due to its relatively small genome size with shorter replication time, as indicated by faster replication and spreading for SFV both *in vitro* and *in vivo* (Figures 2E−2H). Also, SFV was able to prolong survival and even cure mice-bearing neuroblastoma tumors, whereas VV treatment did not have any benefit compared to controls (Figure 3), probably because smaller viruses penetrate and spread better within solid tumors.^27^

Although OVs can mediate the release of TAAs during oncolysis and act as an *in situ* cancer vaccine, they may not be enough to elicit a potent TAA-specific response.^28^ One way to address this issue is to engineer OVs to express one or more tumor antigens, aiming to enhance the TAA-specific immune response.^29^ The insertion of a gene coding for GD2m did not affect any virus replication ability (Figures 2A and 2B). However, neither therapeutic effect nor anti-GD2 antibody was improved by additional expression of the GD2m from either VV-GD2m or SFV-GD2m, in comparison to its parental viruses (Figures 3 and 4), which might be explained by the inability to break self-tolerance.

Several vaccination strategies have been explored to break immune tolerance against self-antigens, one of which is to fuse self-antigen to a xeno-antigen, combined with a strong adjuvant.^30^ NAP is known to be a potent xeno-antigen to boost immune responses against poor antigens.^24^ Others^31^ and we^23^ have shown that arming viruses with NAP has therapeutic benefits in xenograft cancer models. Also, NAP can recruit and activate neutrophils, monocytes, and DCs with a Th1 polarization.^22^^,^^23^ Encouragingly, the addition of NAP to oncolytic VV (VV-GD2m-NAP) significantly enhanced its therapeutic efficacy compared to the unarmed VV-GD2m virus (Figures 5C and 5D). VV-GD2m-NAP treatment was also associated with significantly higher anti-GD2 antibodies (Figure 6A) and induced strong ADCC and higher cytotoxicity to NXS2 tumor cells and NK cell activation (Figure 7). In contrast, no additional benefit was observed for the NAP-armed SFV virus (SFV-GD2m-NAP) in NXS2 tumor-bearing mice (Figures 5E and 5F), and equal levels of anti-GD2 antibodies were induced after SFV-GD2m and SFV-GD2m-NAP treatments (Figure 6B).

The difference between NAP-armed VV and SFV might be due to the intrinsic characteristics of the parental viruses to elicit distinct immune responses.^26^ SFV and VV can induce different cell death modalities as well as the consequent immune response, which ultimately leads to distinct inhibition of tumor growth.^26^ VV carries and encodes many viral proteins such as A52, B15, and K7, suppressing the immune response by inhibiting nuclear factor κB (NF-κB) pathways.^32^ We have shown that VV-infected tumor cells inhibit DC function in T cell priming.^26^ Therefore, arming VV with NAP aids to overcome the virus-mediated inhibitory immune response, resulting in better anti-GD2 antibody response and improved therapeutic efficacy.

On the other hand, we detected a higher anti-SFV-neutralizing antibody response after SFV administration compared to VV (Figures 4C and 4D). SFV infection of tumor cells by nature is very immunogenic and creates an inflammatory milieu supporting DC function.^26^ Hence, further arming SFV with NAP boosted the neutralizing antibody response toward the immune-dominant SFV epitopes rather than the weak immunogen GD2 (Figures 6B and 6D). Similar results have been reported where arming oncolytic vesicular stomatitis virus with CD40L.^33^ Viral proteins are highly immunogenic and more immune dominant than tumor antigens. The immune system naturally tends to focus on viral proteins and ignore the weaker immunogenic tumor antigens.^34^ Taken together, we believe it is the high immunogenicity of SFV protein that distracted immune responses away from generating an anti-cancer response. Further, the provision of an exogenous immune booster only amplified the unwanted anti-virus response.

In conclusion, two distinct OVs (VV and SFV) armed with the same TAA and immune-activation proteins resulted in an opposing immune response and therapeutic benefits. In the case of SFV or similar viruses, which are very immunogenic by nature, the focus of engineering can be directed toward improving direct oncolysis, de-bulking the tumor, releasing TAA, and relying on the virus’s ability to directly induce an anti-tumor immune response. In contrast, for VV or similar viruses, which have slow replication kinetics and by nature have immunosuppressive properties, the focus can be directed toward arming the virus with immune modulators to improve the anti-tumor immune response. Although not considered in the current study, the tumor type and its relative microenvironment can also significantly affect the oncolysis-mediated immune response. The engineering and arming of OVs with immune-modulating agents need careful consideration based on the selected OV and the targeted tumor to achieve the desired therapeutic benefits.

## Materials and methods

### Biosafety level and ethical approval

The Swedish Work Environment Authority has approved the work for genetic modification of SFV/VV (IDs: 202100-2932 v66a14 for laboratory and v67a10 for mice). All experiments regarding modified viruses were conducted under biosafety level 2. The Uppsala Research Animal Ethics Committee has approved all animal studies (N164/15).

### Cell lines and culture conditions

The murine neuroblastoma cell line NXS2 (a gift from Dr. Holger N. Lode, University of Greifswald), the murine melanoma cell line B16-F10 (ATCC CRL-6475; ATCC, Manassas, VA, USA), the human embryonic retinoblast-derived 911 cell line^35^ (Crucell, Leiden, the Netherlands), and human bone osteosarcoma (HOS) cell line (ATCC CRL-1543) were cultured in Dulbecco’s modified Eagle’s medium (DMEM) supplemented with 10% heat-inactivated fetal bovine serum (FBS), 100 units/mL penicillin and 100 μg/mL streptomycin (1% PEST), and 1 mM sodium pyruvate. The baby hamster kidney (BHK) cell line BHK-21 (ATCC CCL-10) was cultured in Glasgow minimal essential medium supplemented with 10% heat-inactivated FBS, 20 mM HEPES, and 10% tryptose phosphate broth (BD Biosciences, Franklin Lakes, NJ, USA). All cells were cultured in a humidified incubator with 5% CO_2_ at 37°C. All components and culture medium were from Thermo Fisher Scientific (Uppsala, Sweden).

### Virus construction and production

All VVs used in this study were based on the VV-dTK strain (a gift from Dr. Bernard Moss, National Institute for Allergy and Infectious Diseases, NIH) and were amplified and purified as described previously.^36^^,^^37^ The GD2m epitope expression cassette was synthesized by GenScript (Piscataway, NJ, USA), which consists of Rluc and GD2m D (MD) with a c-Myc tag, separated by self-cleaving peptide T2A and P2A, respectively. The transgene expression was driven by the vaccinia 7.5k early/late promoter element. VV-GD2m and VV-GD2m-NAP (Figure 1A) were generated by homologous recombination of the GD2m or GD2m-NAP cassette into the *TK* gene site of VV-dTK using shuttle plasmids. All VVs were generated, amplified as purified, as previously reported.^38^^,^^39^

All cloning of SFV A7(74) (SFVA7/74) vectors was designed based on established plasmid pCMV-A7(74)-2SG.^3^ The GD2m-expressing cassette or in combination with NAP (GD2m-NAP) was cloned behind the second subgenomic promoter to generate pCMV-A7/74-GD2m and pCMV-A7/74-GD2m-NAP. Viruses were generated, amplified, and purified according to a previous description^3^ and designated SFV-GD2m and SFV-GD2m-NAP (Figure 1B). An unmodified strain A7(74) was designated SFV. The SFV-GFP virus, expressing GFP, was used for anti-SFV antibody detection.

Both SFV and VV titers were based on a plaque-forming unit (PFU). All of the MOIs used in this study were based on PFU per cell.

### Cell cytotoxicity assay

NXS2 cells were seeded into 96-well plates (1 × 10^4^ cells/well) and infected with VVs at MOIs ranging from 100 to 0.01. Cell viability was measured using a cell viability kit (CellTiter 96 Aqueous One Solution Cell Proliferation Assay Kit; Promega, Nacka, Sweden) at 96 h after infection. The relative cell viability was calculated by using the ratio between the average absorbance for viral-infected cells and the average for non-infected cells. The experiments were performed in triplicate and repeated three times.

NXS2 cells (1 × 10^4^ cells/well) were infected with SFVs at MOIs ranging from 10 to 0.001. Cell viability was measured by using alamarBlue viability reagent (Thermo Fisher Scientific) 72 h after infection. Absorbance of alamarBlue reagent solution was read at excitation at 530 nm and emission at 590 nm on a Synergy HTX Multi-Model Multiplate Reader (BioTek Instruments, Winooski, VT, USA). Cell viability was reported as the percentage of viable cells normalized to non-infected cells. The experiments were performed in triplicate and repeated three times.

### *In vivo* studies

Female, 6- to 8-week-old A/J mice (Taconic, Silkeborg, Demark) were s.c. implanted with NXS2 cells (1 × 10^6^ cells in 100 μL Dulbecco’s PBS [DPBS]) in the right hind flank. The mice were treated i.t. with DPBS (50 μL), VVs (1 × 10^8^ PFU in 50 μL DPBS), or SFVs (1 × 10^7^ PFU in 50 μL DPBS), 7 days post-tumor inoculation when the tumors were palpable (size approximately 50 mm^3^). Blood samples were harvested 14 days after treatment to analyze the level of anti-GD2 and anti-virus antibody.

#### Survival analysis

The animals were monitored individually for tumor growth until the tumor volume exceeded the study endpoint volume (EPV; 1000 mm^3^); tumor size was calculated using the ellipsoid volume formula,

$$Tumor\;volume=\frac{Length\times Width^{2}\times \pi }{6}$$

The time to endpoint (TTE) for each mouse was calculated as TTE = (log [EPV] – b)/m, where the constant b is the intercept, and m is the slope of the line obtained by linear regression of time. A log-transformed tumor growth dataset is comprised of the first measured tumor volume when EPV was exceeded and three consecutive measured tumor volumes immediately prior to the attainment of EPV. Survival curve was generated based on the TTE value using the Kaplan-Meier method and compared using the log-rank (Mantel-Cox) test.

### Immunohistochemistry

To evaluate i.t. replication of viruses, tumor was collected 2 days after virus injection. Paraffin-embedded tumor samples were sliced into 5 μm sections and deparaffinized. Antigen revival was performed by heating the slides at 121°C for 20 min in antigen revival solution (Vector Laboratories, Burlingame, CA, USA). Sections were blocked with goat serum (1:100; Vector Laboratories). For VVs, sections were stained with rabbit anti-VV A27L antibody (1:2,000; Abcam, Cambridge, UK) followed by staining with goat anti-rabbit-AF647 (1:2,000; Invitrogen, Carlsbad, CA, USA); nuclei were stained with Hoechst 33342 (1:2,000; Invitrogen). For SFVs, sections were stained with rabbit anti-SFV structural protein polyclonal antibody (1:3,000; a kind gift from Dr. Ari Hinkkanen, University of Eastern Finland) and goat anti-rabbit horseradish peroxidase (HRP; 1:1,000; Invitrogen). Signals were visualized with ImmPACT DAB chromogen (Vector Laboratories). The slides were then counterstained with hematoxylin.

### PFU virus titration

To evaluate virus replication *in vivo*, tumor was collected 3 days after virus injection. Viral titers were determined by plaque assays using 100% confluent, either BHK cells (for SFV viruses) or HOS cells (for VVs) in a 6-well plate. Tumors were lysed to extract virus particles, and a 10-fold dilution series of viruses was added to the cell monolayer and incubated for 1 h. Then the monolayer was washed with PBS and overlaid with DMEM containing 0.8% low-melting agarose (Invitrogen). After 72 h incubation, the cells were stained with 1% crystal violet (Sigma, Munich, Germany), and visible plaques were counted. The progeny virus titer from tumor lysate was normalized to tumor weight and represented as PFU/gram of tumor.

### Detection of GD2m peptide

To analyze GD2m peptide expression in the tumor, the tumor was collected from NXS2 tumor-bearing mice, 2 days after OV treatment. For VV-based treatment, paraffin-embedded tumors were sliced into 6 μm sections. Deparafinized sections were stained with mouse anti-c-Myc antibody (MA1-980; Thermo Fisher Scientific), followed by goat anti-mouse-AF488 antibody (1:2,000; Jackson ImmunoResearch Laboratories, Täby, Sweden). The sections were imaged by a Zeiss AxioImager microscope (Zeiss, Oberkochen, Germany). For SFV-based treatment, single cells were isolated from harvested tumors and stained with mouse anti-c-Myc antibody, followed by goat anti-mouse-AF647 (1:2,000; Invitrogen). Stained cells were analyzed by flow cytometry BD FACSCanto II (BD Biosciences, Franklin Lakes, NJ, USA).

To verify that the GD2m is structurally mimicking the GD2 antigen, B16-F10 was infected with VV-GD2m at MOI 1. Cells were collected 48 h post-infection, fixed with 4% paraformaldehyde, and stained with anti-GD2 antibody (14G2a; BD Pharmingen, Heidelberg, Germany) at 1 μg/mL Tris-buffered saline (TBS). After incubation with goat anti-rabbit-AF647 (1:2,000; Invitrogen), the MD expression was analyzed by FACSCanto II (BD Biosciences).

### Detection of GD2-specific IgG antibody in sera

To determine the anti-GD2 serum response, GD2 (Sigma) was coated onto an ELISA plate (96 wells, flat bottom; Sartstedt, Germany) at 50 ng/50 μL in methanol per well and dried by evaporation. The precoated plates were blocked with 1% bovine serum albumin (BSA) (Sigma) in PBS containing 0.05% Tween 20 (PBST) (Invitrogen) for 1 h at room temperature (RT). The serum samples (1:100 dilution) were added, and the plates were incubated for 2 h, RT. After washing (5×, PBST), the secondary antibody goat anti-mouse IgG-HRP (Santa Cruz Biotechnology, Santa Cruz, CA, USA) was added (1:2,000, 1 h, RT) to the plates, followed by adding substrate reagent 3,3′,5,5′-tetramethyl-benzidine (TMB; Thermo Fisher Scientific, Carlsbad, CA, USA). After adding 0.2 M sulfuric acid, the plates were analyzed at absorbance 450 nm by an ELISA plate reader (Bio-Rad, Munich, Germany).

### Detection of anti-virus neutralization assay

To determine the neutralizing anti-virus antibody titer, mice serum was collected 14 days after treatment. The serum was serially diluted ranging from 1:50 to 1:400 (VV) or 1:100 to 1:800 (SFV). The diluted serum was mixed with either VV-GD2m or SFV-GFP (MOI 10 to infect 911 cells seeded at 1 × 10^4^ per well in a 96-well plate). After incubating at 37°C for 1 h, the virus-serum mixture was used to infect pre-seeded 911 cells. At 18 h post-infection, the cells were analyzed for transgene expression. For VV-GD2m, Rluc activity (as an indicator of VV-infected cells) was determined using the Rluc Reporter Assay System (Promega) according to the manufacturer’s protocol. For SFV-GFP, the cells were analyzed by FACS to measure the percentage of SFV-infected cells (GFP was used as an indicator of SFV-infected cells). Single-cell suspensions were fixed in 1% paraformaldehyde and analyzed by using a Cytoflex LX Flow Cytometer (Beckman Coulter, Bromma, Sweden). The neutralization capacity of serum samples was calculated with the following formula:

$$Neutralization=1-\frac{Value\left(Sample\right)}{Value\left(Naive\right)}$$

### Assessment of ADCC

ADCC assays were performed in round-bottom, tissue-culture-treated polystyrene 96-well plates. All assays were performed in R10 culturing medium containing 10 international units (IU) IL-2.

For an antibody-mediated cytotoxicity assay, heated-inactivated plasma (56°C for 30 min) was diluted (1:50, 1:100, and 1:200) and added into wells. Target cells (10^4^ NXS2-luc cells, which express firefly luciferase as an indicator of target cell viability) and effector cells (10^6^ murine fresh isolated splenocytes) were added to the plasma. After incubation for 24 h, luciferase activity was measured. Relative cell viability was calculated by normalizing relative light units (RLUs) from samples against the RLUs from wells containing only target cells. The wells containing target cells, effector cells, and plasma isolated from naive mice were set as 100% RLU. The percentage of specific killing was calculated with the following equation:

$$Relative\;killing=RLU\left(Naive\right)-RLU\left(Sample\right)$$

For antibody-mediated NK cell activation, heated-inactivated plasma was 1:100 diluted and added into wells, together with target cells (10^4^ NXS2 cells) and effector cells (10^6^ murine fresh isolated splenocytes). In addition, anti-mouse CD107a-PE/Cy7 (1D4B clone; BioLegend, Täby, Sweden), 5 mg/mL brefeldin A (BD Biosciences), and 5 mg/mL monensin (BD Biosciences) were added and incubated for less than 12 h at 37°C, 5% CO_2_. Cells were then stained with Fixable Viability Stain 700 (BD Biosciences), anti-mouse CD45-BV510 (30-F11 clone; BioLegend), CD3-fluorescein isothiocyanate (FITC; 17A2 clone; BioLegend), and NKp46-BV421 (29A1.4 clone; BD Biosciences) for 30 min at RT in the dark. Then the cells were permeabilized using the True-Nuclear Transcription Factor Buffer Set (BioLegend). These permeabilized cells were then stained with IFN-γ-PE (XMG1-2 clone; BioLegend) for 30 min at RT in the dark. Samples were analyzed by using the Cytoflex LX Flow Cytometer (Beckman Coulter).

### Statistical analysis

Statistical analysis was performed by using GraphPad Prism software 6 (La Jolla, CA, USA). One-way ANOVA test was used for statistical comparison of means between more than two experimental groups in one experiment. Statistical comparison of Kaplan-Meier survival curves was performed by log-rank (Mantel-Cox) test.

## Supplemental information

Document S1. Supplemental materials and methods and Figures S1 and S2

Document S2. Article plus supplemental information
