# Supplementary material for: Concurrent expression of HP-NAP enhances antitumor efficacy of oncolytic vaccinia virus but not for Semliki Forest virus
Source: Mol Ther Oncolytics. 2021 May 5;21:356–66. doi: 10.1016/j.omto.2021.04.016 (PMC8182386; doi:10.1016/j.omto.2021.04.016)
Supplement: Document S1. Supplemental materials and methods and Figures S1 and S2 [file mmc1.pdf]

OMTO, Volume *21*

Supplemental information

Concurrent expression of *HP-NAP* enhances antitumor efficacy of oncolytic vaccinia virus but not for Semliki Forest virus

Jing Ma, Chuan Jin, Matko Čančer, Hai Wang, Mohanraj Ramachandran, and Di Yu

Supplementary Material and Methods

Adenovirus construction and production

All recombinant replication-deficient adenoviral vectors used in this study were based on human adenovirus serotype 5 (Ad5) and were constructed by using AdEasy technology[1]. The GD2 epitope mimotope expression cassette (GD2m) was synthesized by GenScript (Piscataway, NJ) and insert into the shuttle vector to generate pShuttle(CMV-GD2m). The Ad5 backbone vector pAdEasy(E3) was used to construct Ad5(GD2m) through homologous recombination.

Higher titer recombinant adenovirus was produced in 911 cells by several rounds of amplification and were purified by CsCl gradient ultracentrifuge at 25,000 rpm at 4°C for 2h, dialyzed against a dialysis buffer (10 mM Tris-HCl (pH 7.9), 2 mM $MgCl_2$ and 4% w/v sucrose), aliquoted and stored at -80°C. Virus titers were determined as encapsidated viral genome (evg) by quantitative PCR on 911 cells, as described earlier[2].

Animal studies

Adenovirus-vaccination study:

Female A/J mice (Taconic, Demark), 6-8 weeks old, were injected intraperitoneally with Ad-GD2m (1×10^8 evg in 100µl DPBS) or PBS (100µl) on day 0 and 18. The vaccinated mice were subcutaneously implanted with NXS2 cells (1×10^6 cells in 100µl DPBS) on day 30 in the right hind flank (Supplementary Figure S1A).

VV-NAP treatment study:

Female A/J mice (Taconic, Demark), 6-8 weeks old, were subcutaneously implanted with NXS2 cells (1×10^6 cells in 100µl DPBS) in the right hind flank. Seven days after tumor inoculation (tumor size was around 50 mm^3), the mice were treated intratumorally (i.t.) with DPBS (50µl), VV-GD2m, VV-NAP and VV-GD2m-NAP (1×10^8 PFU in 50µl DPBS).

Survival analysis: The animals were monitored individually for tumor growth until the tumor volume exceeded the study endpoint volume (EPV, 1000 mm3); tumor size was calculated using the ellipsoid volume formula= length×width2×π/6. The time to endpoint (TTE) for each mouse was calculated as TTE= [log (EPV)–b]/m, where the constant b is the intercept and m is the slope of the line obtained by linear regression of time. A log-transformed tumor growth data set, which comprised of the first measured tumor volume when EPV was exceeded and three consecutive measured tumor volume immediately prior to the attainment of EPV. Any animal determined to have died from treatment-related causes was assigned a TTE value equal to the day of death. Any animal that died from non-treatment–related causes was excluded from the analysis. Survival curve was generated based on the TTE value using the Kaplan-Meier method, and compared using the log-rank (Mantel-Cox) test.

References

1. He, TC, Zhou, S, da Costa, LT, Yu, J, Kinzler, KW, and Vogelstein, B (1998). A simplified system for generating recombinant adenoviruses. *Proc Natl Acad Sci U S A* **95**: 2509-2514.

2. Ramachandran, M, Yu, D, Wanders, A, Essand, M, and Eriksson, F (2013). An infection-enhanced oncolytic adenovirus secreting H. pylori neutrophil-activating protein with therapeutic effects on neuroendocrine tumors. *Mol Ther* **21**: 2008-2018.

Supplementary Figure S1

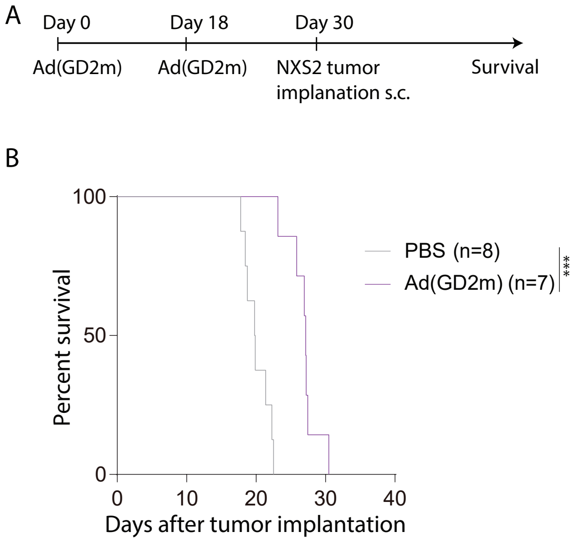

Supplementary Figure S1. Vaccination with GD2m-expressing human Adenovirus (Ad-GD2m) prolonged mice survival when challenged with NXS2 neuroblastoma. A) Experimental scheme for Ad-GD2m vaccination and NXS2 tumor cell challenge. Female A/J mice were intraperitoneally injected with Ad-GD2m or PBS on day 0 and 18, and subcutaneously implanted with NXS2 cells (1×10^6 cells in 100µl DPBS) on day 30 in the right hind flank. **B**) Kaplan-Meier survival curves were shown and compared using log-rank test (***: P<0.001).

Supplementary Figure S2

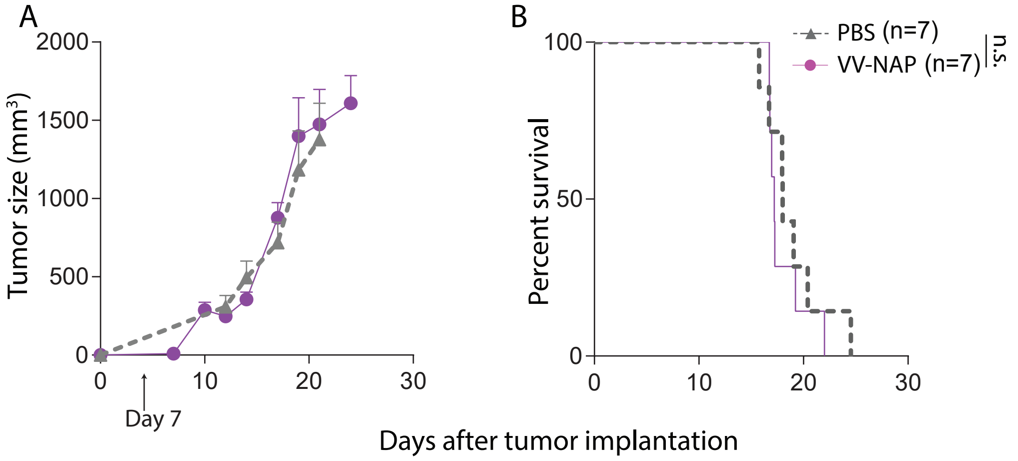

Supplementary Figure S2. VV-NAP exhibited no additional therapeutic benefit compared to either VV-GD2m or PBS vehicle control. A-B) A/J mice harboring murine neuroblastoma NXS2 tumor were intratumorally treated with either PBS, or VV-NAP virus on day 7 after tumor implantation. (**A**) The tumor sizes (mean+SEM) and (**B**) Kaplan-Meier survival curves were shown. Survival curves were compared using log-rank test. (**: *P*<0.01; ***: *P*<0.001).
